# Identification of resilient individuals and those at risk for performance deficits under stress

**DOI:** 10.3389/fnins.2015.00328

**Published:** 2015-09-16

**Authors:** Brent D. Winslow, Meredith B. Carroll, Jonathan W. Martin, Glenn Surpris, George L. Chadderdon

**Affiliations:** Design Interactive, Inc.Orlando, FL, USA

**Keywords:** stress, simulation, behavior, autonomic reactivity, resilience

## Abstract

Human task performance is affected by exposure to physiological and psychological stress. The ability to measure the physiological response to stressors and correlate that to task performance could be used to identify resilient individuals or those at risk for stress-related performance decrements. Accomplishing this prior to performance under severe stress or the development of clinical stress disorders could facilitate focused preparation such as tailoring training to individual needs. Here we measure the effects of stress on physiological response and performance through behavior, physiological sensors, and subjective ratings, and identify which individuals are at risk for stress-related performance decrements. Participants performed military-relevant training tasks under stress in a virtual environment, with autonomic and hypothalamic-pituitary-adrenal axis (HPA) reactivity analyzed. Self-reported stress, as well as physiological indices of stress, increased in the group pre-exposed to socioevaluative stress. Stress response was effectively captured via electrodermal and cardiovascular measures of heart rate and skin conductance level. A resilience classification algorithm was developed based upon physiological reactivity, which correlated with baseline unstressed physiological and self-reported stress values. Outliers were identified in the experimental group that had a significant mismatch between self-reported stress and salivary cortisol. Baseline stress measurements were predictive of individual resilience to stress, including the impact stress had on physiological reactivity and performance. Such an approach may have utility in identifying individuals at risk for problems performing under severe stress. Continuing work has focused on adapting this method for military personnel, and assessing the utility of various coping and decision-making strategies on performance and physiological stress.

## Introduction

Human behavioral task performance is affected by exposure to psychological and physiological stress in a dose-dependent manner, as originally posited more than a century ago (Yerkes and Dodson, 1908). Moderate stress can improve cognitive performance, via catecholamine-induced increases in brain glucose utilization (Cousijn et al., 2012), modulation of hippocampal activity (Weerda et al., 2010), or other mechanisms. However, severe stress can reduce fine motor performance (Lieberman et al., 2005), attention (McHugh et al., 2010), and cognitive function (van Wingen et al., 2012) due to biological and neural mechanisms. Given high stress occupations such as military service or emergency response, there is a need to understand how elevated levels of stress impact performance in order to effectively train individuals to perform successfully in the field (van Wingen et al., 2012). Further, there is a need to identify individuals at risk for stress-related problems with performance or cognition prior to actual performance under severe stress or the development of clinical stress disorders such as major depressive disorder (MDD), post-traumatic stress disorder (PTSD), or suicide ideation and attempt, as prolonged exposure to stress increases the risk of such conditions (Hoge et al., 2004). Available evidence suggests that experiencing severe stress has a direct impact on PTSD development, and that the association between traumatic stress and PTSD symptoms can be moderated by an individual's level of resilience to stress (Lee et al., 2014). Recent increases in military rates of PTSD (Baker et al., 2012) and suicide in the wake of the conflicts in Iraq and Afghanistan (Milliken et al., 2007), illustrate the need for training to prepare individuals to recognize and cope with severe stress both during performance and in the aftermath. Finally, the ability to identify those individuals who are more susceptible to a prolonged stress response prior to a significantly stressful event, could lead to a focused effort to better prepare those most in need.

The ability to measure individual differences in physiological response to stressors may facilitate the identification of individuals who are not resilient to stress, that is, more susceptible to a severe stress response, and less likely to recover from such a response. Associated with stress response and recovery is the concept of resilience, a broad term defining a process of coping with or overcoming exposure to adversity or stress (Meredith et al., 2011). For our purposes, resilience is defined by behavioral and physiological changes in response to, and recovery following exposure to stress, specifically, whether an individual remains unresponsive to a stressor, has a significant reaction to a stressor, then recovers to pre-stressor state, or has a significant reaction to a stressor but does not recover to pre stressor state). Typically, an individual's resilience is qualitatively defined either by one of a number of self-report scales (Wagnild and Young, 1993; Block and Kremen, 1996; Connor and Davidson, 2003), or quantified by behavioral changes such as the development of MDD, PTSD, or suicide. However, recent studies suggest that there may be ways to identify more objective measures such as physiological markers of resilience. For instance, the brains of individuals diagnosed with clinical stress disorders exhibit epigenetic effects on critical players in the stress response, including decreased levels of glucocorticoid receptors (GRs) in various brain areas (McGirr et al., 2010; Flinn et al., 2011). Early life stress has also been shown to significantly affect GRs and other sex-specific hormone receptors (Bogdan and Hariri, 2012; Burghy et al., 2012; Wei et al., 2013). Although human brain GRs cannot currently be assessed noninvasively, indirect individual-level assessment and correlation may be possible via physiological and behavioral changes following stress induction. Measures such as non-stressed cortisol levels and cortisol reactivity may provide insight into an individual's susceptibility to severe stress response (Gunnar et al., 2001; Burghy et al., 2012; Owens et al., 2014).

A number of recent studies have focused on factors which augment or diminish resilience in individuals (Parker and Maestripieri, 2011; Obradovic, 2012). However, to date, reports on human resilience capacity have focused on long-term trajectories (weeks to months), with little or no focus on whether short term resilience trajectories (minutes to hours) can be assessed and whether significant changes to resilience could be achieved by short-term interventions or training scenarios (Norris et al., 2009; Peres et al., 2011).

The primary objective of this experimental study was to measure individual stress responsivity and recovery from acute stressors and the impact stress had on performance in military-relevant training scenarios using behavior, physiological inputs, and subjective ratings in order to identify measures predictive of individual resilience. Stress was induced using a combination of video-games based stressors and socioevaluative stress as previous groups have shown that the induction of physiological stress during video game-based scenarios is difficult (Biondi and Picardi, 1999), but that the use of socioevaluative stress protocols can induce significant physiological stress (Dickerson and Kemeny, 2004). A control group received the video-game based stressors only to determine their effectiveness compared to socioevaluative stress. It was hypothesized that a significant stress response would occur in both conditions, with the greatest stress response during socioevaluative stress, and that inputs from physiology, behavior, and self-report measures could be used to quantify and predict individuals' resilience to stress in order to identify individuals at risk for stress reactivity disorders. In the current study, baseline subjective stress ratings and physiological data were compared and correlated against data obtained under stressful conditions to develop algorithms to assess and predict individual resilience to stress.

## Materials and methods

### Participants

All methods involving participants were approved by an independent Institutional Review Board (Copernicus Group, Durham, NC). Forty novice participants [33 male; average age 25.5 ± 4.0 (SD) years] completed and received payment of $100 USD for participation in the study, which lasted approximately 3 h. All participants were recruited from the community and met minimum requirements including age (18–35), normal visual acuity, and no medical conditions such as endocrine disorders.

### Experimental procedure

**Figure 1:**
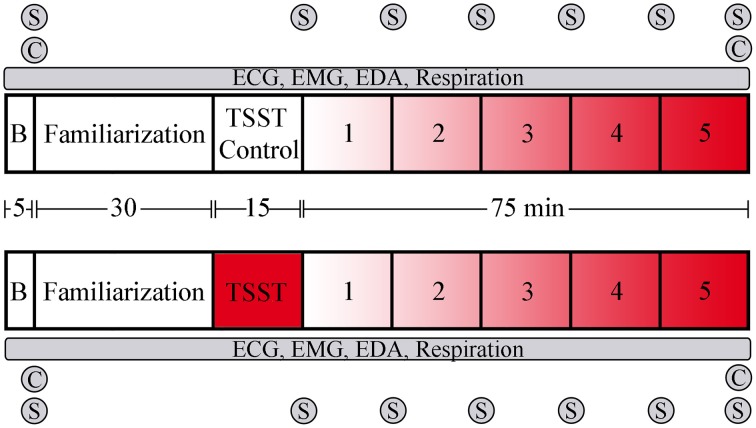
**Overview and timeline of the experimental design**. The upper portion represents the control group that did not receive socioevaluative stress prior to scenario performance, while the lower portion represents the group that received the TSST. Following baseline physiological recording and training in the virtual environment, participants went through the TSST or control task. Task presentation followed immediately, with task order counter-balanced via a Latin squares method. B, baseline period; C, saliva sample for cortisol measurement; S, STAI administration. Red shading indicates level of stressor inclusion.

The experimental procedure is overviewed in Figure 1. Participants arrived between 8:00 and 8:30 a.m., provided written, informed consent, completion of a demographics questionnaire, and the state portion of the state-trait anxiety inventory [STAI] (Spielberger et al., 1983), previously shown to have a high test-retest reliability with situational stress (Rule and Traver, 1983). Wireless physiological sensors were then placed on the participants, followed by a 5-min recording of baseline physiological activity while participants remained seated. Participants were instructed prior to arrival to abstain from eating, drinking, and smoking for 1 h prior to arrival. Participants rinsed with water, then provided a saliva sample for cortisol measurement via passive drool, which was immediately frozen. Following baseline procedures, participants were familiarized with the virtual environment (VE) controls, and keyboard commands, without exposure to any virtual stressful components. Participants were then randomly assigned to either the experimental group, which received the Trier Social Stress Test [TSST; (Kirschbaum et al., 1993)], or the control group. The TSST was used as an external socio evaluative stressor prior to task performance, consisting of 5 min each of: anticipatory stress; oral presentation; and mental arithmetic. The control group received a placebo version of the TSST (Het et al., 2009), which contained the same factors except for the psychosocially stressful components. In the placebo version, participants read a document out loud for the public speaking portion of the TSST and counted forward by 2 for the mental arithmetic portion. Performance was then assessed while all participants performedfive complex military-relevant scenarios within Virtual Battlespace 2 (VBS2, Bohemia, Orlando FL), a VE used for military training. Scenarios were created in VBS2 to simulate a tactical military environment with specific mission objectives, time requirements, and consequences depending on the course of action a participant pursued. Scenarios 1–5 were designed to sequentially increase in stress by varying the number of stressors and stressor characteristics such as novelty, predictability, and controllability. Each scenario also includes 5 specific decision-making events triggered by timing or participant location. Scenario 1, designed to be the lowest stress scenario, consists of the participant following a person of interest (POI) through a virtual town during daylight hours and noting his activities. Decisions in this scenario included when to follow the POI, the distance to maintain, and reacting to the POI's actions. Scenario 2 is a night mission in which the participant must set off a demolition charge, enter a restricted area without being observed, obtain information, and evacuate. Decisions in this scenario included target identification and prediction of enemy movement. Scenario 3 is a night mission in which the participant must avoid enemy detection and set a series of demolition charges prior to evacuating the area. Decisions in this scenario included when to detonate the demolition charges, target identification, and maintaining sufficient distance from enemies. In scenario 4 the participant's helicopter has crashed in enemy territory at night and the participant must evacuate under heavy fire. Decisions in this scenario included target identification and whether to engage or avoid enemies. In scenario 5, designed to be the highest stress scenario, the participant must perform long range fire during daylight hours in enemy territory under fire. Decisions in this scenario included target identification, and whether to engage or avoid enemies. Simulation-based stressors included limited visual perception, sudden noise exposure (Hockey, 1970; Rhudy and Meagher, 2001), equipment failures, and receiving enemy fire, as well as cognitive stressors such as time pressure, and emotion induction procedures (Bouchard et al., 2010; Cousijn et al., 2012), including the presentation of dead combatants, soldiers, and civilians (Dickerson and Kemeny, 2004). The stressfulness of each scenario was independently coded by 3 unbiased observers. VBS2 scenarios were presented on a PC running on a Pentium i5 quad core processor. Physiological measures were captured throughout the baseline phase, socio evaluative stress phase and the VBS2 scenarios. At the end of the socio evaluative stress phase and each scenario, participants completed an additional STAI. Following all scenarios, participants provided a second saliva sample, and were debriefed and paid for their participation.

### Measurements

Physiological measures, subjective stress measures, and performance measures were used to assess individual stress response and resilience.

#### Physiological measurements

Participants were fitted with a 3-lead electrocardiogram (ECG) with bandlimits set between 1 and 35 Hz, a electromyogram [EMG; (Jensen et al., 1993)] on the upper left trapezius, with bandlimits set between 10 and 500 Hz, a respiration strap to record changes in thoracic expansion with bandlimits set between DC and 1 Hz, and palmar electrodermal activity (EDA) on the 4th and 5th fingers of the non-dominant hand with bandlimits set between DC and 10 Hz. All physiological data was sampled at 500 Hz and wirelessly sent to an MP-150 system running AcqKnowledge software (Biopac Systems, Goleta CA). Gain was set on EMG and ECG channels to 2000. The root mean square (rms) was calculated by averaging over 100 EMG samples. The EDA data was run through digital bandpass filters at 1 and 0.05 Hz, followed by thresholding between 0.05 and 0.051 μS to identify electrodermal responses (EDR), which were quantified at a per minute rate. Heart rate was calculated from the R-R interval from the ECG, with intervals < 40 and >180 bpm excluded from the analysis. Respiratory rate and respiratory sinus arrhythmia (RSA) was calculated using the peak valley method outlined in Grossman et al. (1990). Briefly, the ECG signal was used to define R-R intervals, and the respiration strap was used to define a respiratory period. RSA was calculated as the difference between the maximum and minimum heart rate within a respiratory cycle. Frequency-domain heart rate variability (HRV) was obtained using Fast Fourier Transform, followed by an analysis of low frequency (LF) power (0.04–0.15 Hz), indicative of sympathetic activity, and high frequency (HF) power (0.15–0.4 Hz), indicative of vagal activity (Jaffe et al., 1993). Temporal-domain HRV was calculated as the standard deviation of the R-R intervals (SDNN) from the ECG over >5 min. Root mean square (rms) of the EMG signal was averaged over 100 samples (200 ms). Salivary cortisol was measured by standard ELISA (Salimetrics, Carlsbad CA; intra-assay CV = 4.5%, inter-assay CV = 5.8%).

#### Subjective stress measurements

Perceived stress was measured at baseline and immediately following each task using the state portion of the STAI (Spielberger et al., 1983). The STAI consists of 20 statements, and participants rank how closely the statement matches how they feel currently. Scores range from 20 to 80, with higher scores indicating the presence of higher levels of stress. The presence of external stressors was assessed via questionnaire by asking participants whether they experienced a recent bereavement (e.g., loss of loved one, home, job, etc.), and whether they had significant academic exams in the next 2 weeks or upcoming surgery/medical procedure.

#### Performance measurements

Performance scores were calculated based upon data obtained in VBS2 including duration of scenario, mission objectives being met, number of times the participant was shot during a scenario, and whether non-enemies were fired on by the participant. The performance scores reflected the degree to which the participant achieved the goals set forth in the mission while minimizing mission time, shots taken and civilians killed. Performance scores had a maximum value of 10 and a minimum value of 0.

### Data analysis and statistics

First, a series of mixed within, between groups repeated measures Analysis of Variance (ANOVAs) were used to analyze differences in average values throughout the experiment for self-reported stress, performance, EDR, cardiovascular measures and EMG, with a within group factor of timepoint (baseline, TSST, and the 5 VBS2 scenarios), and a between group factor of condition (control, *n* = 20; experimental, *n* = 19). Between groups, independent *t*-tests were used to establish differences between groups at a given trial. All statistical testing was done in SPSS software version 18.

To assess individuals' resilience to stress, a physiological stress score was defined for the experimental group using a combination of heart rate and skin conductance level (SCL), normalized by the mean control group values. Subjects were grouped by whether their heart rate and skin conductance remained unresponsive to the stressor (resistant-trend; baseline to TSST percent change ≤ 5%), rose with the stressor, then fell back to baseline values (resilient-trend; baseline to TSST percent change > 5% and baseline to scenario percent change ≤ 5%), rose with the stressor and fell back to stress levels higher than baseline values (recovery-trend; baseline to TSST percent change > 5% and baseline to scenario percent change > 5%), or rose with the stressor and continued to rise (dysfunctional-trend, not observed in this data). (Norris et al., 2009) provided the basis for our definitions of the trends.

To determine if there was a predictive relationship between individual baseline measures and stress responsivity during the experiment, a classifier was developed to map the features of pre-trial cortisol levels and baseline STAI to one of the resilience-trend classes defined above (recovery, resilient, resistant). Supervised learning algorithms from the Python machine learning library scikit-learn (Pedregosa et al., 2011) were trained using the resilience group-labeled 2-feature exemplars for each subject. Classification methods tested included the following: stochastic gradient descent linear discrimination, logistic regression, perceptron learning, linear kernel support vector machines, 1 nearest-neighbor, Gaussian naïve Bayes, and Gaussian mixture models. Due to the small sample size (*N* = 16), the entire non-discarded data set was used to learn the decision boundaries of the candidate classifiers.

## Results

### Demographics

The sociodemographic characteristics of the participants in this study are presented in Table 1. All were healthy, non-smokers without any current medical conditions. The age of participants ranged from 21 to 35, with a mean of 25.5 years and approximately 6 h per week of video game use. Most were undergraduate students, with 12.5% experiencing significant life stressors including recent bereavement, or major upcoming examinations.

**Table 1:** **List of sociodemographic factors of study sample**.

	**Study sample % (n)**
Gender	
Male	82.5 (33)
Female	17.5 (7)
Age Group	
21–25	55 (22)
26–30	27.5 (11)
31–35	17.5 (7)
Education	
High School Diploma	27.5 (11)
Some College/University	67.5 (27)
University Degree	5 (2)
**Hormonal Contraceptive**	10 (4)
**Life Stress**	12.5 (5)
Hours of video game playing per week	
Under 3	40.0 (16)
3–9	37.5 (15)
10–16	17.5 (7)
17 or more	5.0 (2)

### Self-reported stress and performance

**Figure 2:**
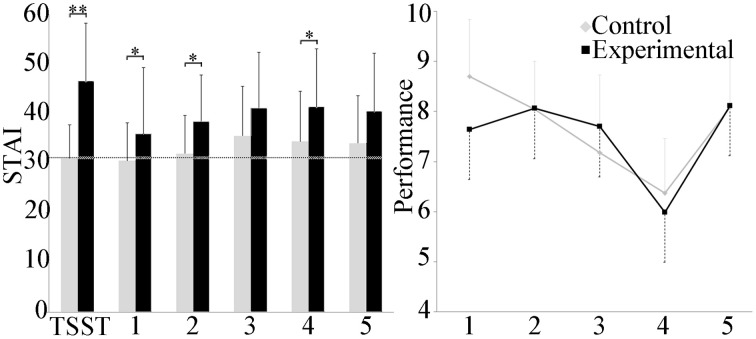
**Group differences in the STAI and performance score**. The experimental group reported significantly higher self-reported stress after the TSST and scenarios 1, 2, and 4 than the control group. Average baseline for the cohort is shown in the dashed line. Performance in scenario decreased linearly with the exception of scenario 5 and was similar for both groups. ^*^p ≤ 0.05; ^**^p ≤ 0.001.

Self-reported stress levels are presented in Figure 2. Between groups, the experimental group reported a significant increase in stress as compared to the control group in scenarios 1, 2, and 4 (p ≤ 0.05) and during the TSST (p ≤ 0.001). Within groups, repeated measures ANOVA showed that participants in the control group reported stress levels throughout the experiment that did not differ from baseline with the exception of the 3rd scenario (p ≤ 0.05). In the experimental group, perceived stress increased during the TSST portion of the experiment, as well as during scenarios 3 and 4 (p ≤ 0.05).

No differences in scenario performance between experimental and control groups were observed. Within groups, scenario performance decreased linearly over the first 4 scenarios in the control group, with the exception of the 5th scenario. Repeated measures ANOVA indicated differences between scenario 4 and scenarios 1, 2, and 5 (p ≤ 0.001), and between scenarios 1 and 3 (p ≤ 0.001). In the experimental group only, repeated measures ANOVA also revealed a difference between scenarios 4 and scenarios 2, 3, and 5 (p ≤ 0.05).

### Electrodermal activity

**Figure 3:**
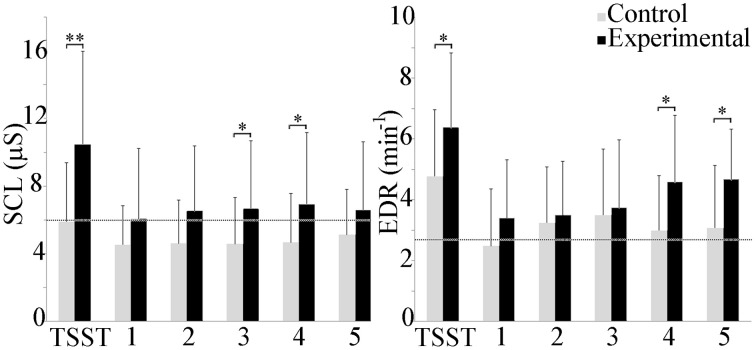
**Group differences in mean SCL and EDR rate**. The experimental group exhibited higher electrodermal activity throughout the experiment. Average baseline scores shown by dotted line. ^*^p ≤ 0.05; ^**^p ≤ 0.001.

Electrodermal activity is shown in Figure 3. Between groups, a statistically significant increase was observed in the experimental group during the TSST, and scenarios 3 and 4 (*p* ≤ 0.05). Mean skin conductance level (SCL) exhibited high variability throughout the study, with a trend toward increasing values in the experimental group. Within groups, repeated measures ANOVA analysis did not detect any differences in the control group, but did identify an increase in the TSST compared to scenario 1 in the experimental group (*p* ≤ 0.001), and between the TSST and the baseline as well as scenarios 2, 3, and 5 (*p* ≤ 0.05).

Minute EDR was higher in the experimental group throughout the course of the experiment. Between group differences were observed during the TSST, and scenarios 4 and 5 (*p* ≤ 0.05). Within groups, repeated measures ANOVAs indicated that EDR during the TSST in the control group was significantly higher than during the baseline and scenarios 1 and 5 (*p* ≤ 0.05). In the experimental group, EDR during the TSST was significantly higher than during the baseline and scenarios 1 and 2 (*p* ≤ 0.001) as well as scenario 3 (*p* ≤ 0.05).

### Cardiovascular response

**Figure 4:**
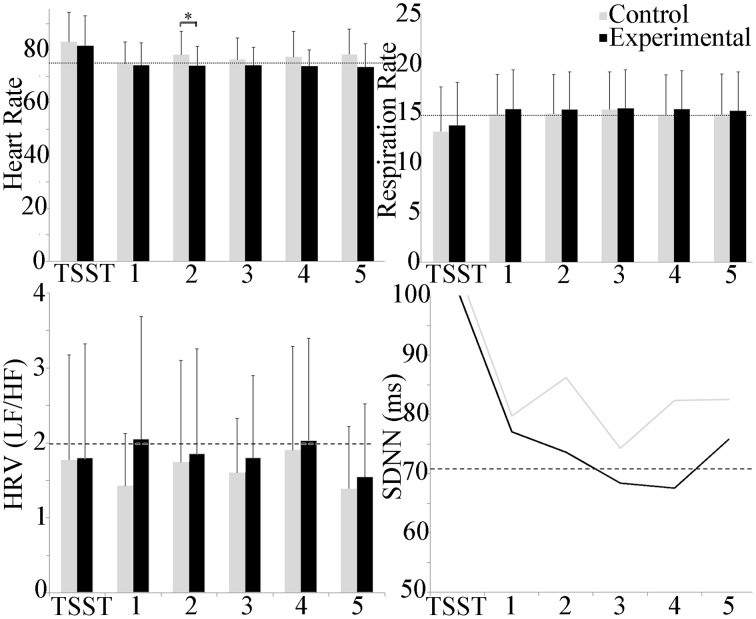
**Group differences in cardiovascular measures during the experiment**. Heart rate trended higher during the TSST in both groups, and throughout in the experimental group. A group difference in heart rate was observed during scenario 2 only. Respiration rate trended higher during scenarios for both groups, and lower during the TSST. LF/HF activity was dominated by sympathetic activity. No group or scenario differences were observed. Temporal domain HRV (SDNN) showed a significant increase in the TSST as compared to all other scenarios for the experimental group. Average baseline scores shown by dotted line. ^*^p ≤ 0.05.

Mean cardiovascular effects are shown in Figure 4. Between groups, heart rate trended higher in the experimental group as compared to control throughout, but only achieved statistical significance during scenario 2 (*p* ≤ 0.05). Within groups, repeated measures ANOVA showed a statistically significant increase in the experimental group between the TSST and scenarios 2, 3, and 4 (*p* ≤ 0.001) and between the TSST and baseline and scenarios 1 and 5 (*p* ≤ 0.05). Respiration rate was higher during the scenarios as compared to baseline, and lower during the TSST for both groups, however, repeated measures ANOVA did not detect differences across scenarios, and *t*-tests did not find differences between groups. The LF/HF ratio, indicative of whether sympathetic (LF) or vagal (HF) activity dominated throughout the experiment, showed a trend toward sympathetic activity throughout, but statistical analysis did not reveal differences between or within groups for both conditions. Temporal domain HRV (SDNN) showed a significant increase between the TSST and the baseline as well as scenarios 2, 3 (*p* ≤ 0.05) and 4 (*p* ≤ 0.001) within the experimental group.

**Figure 5:**
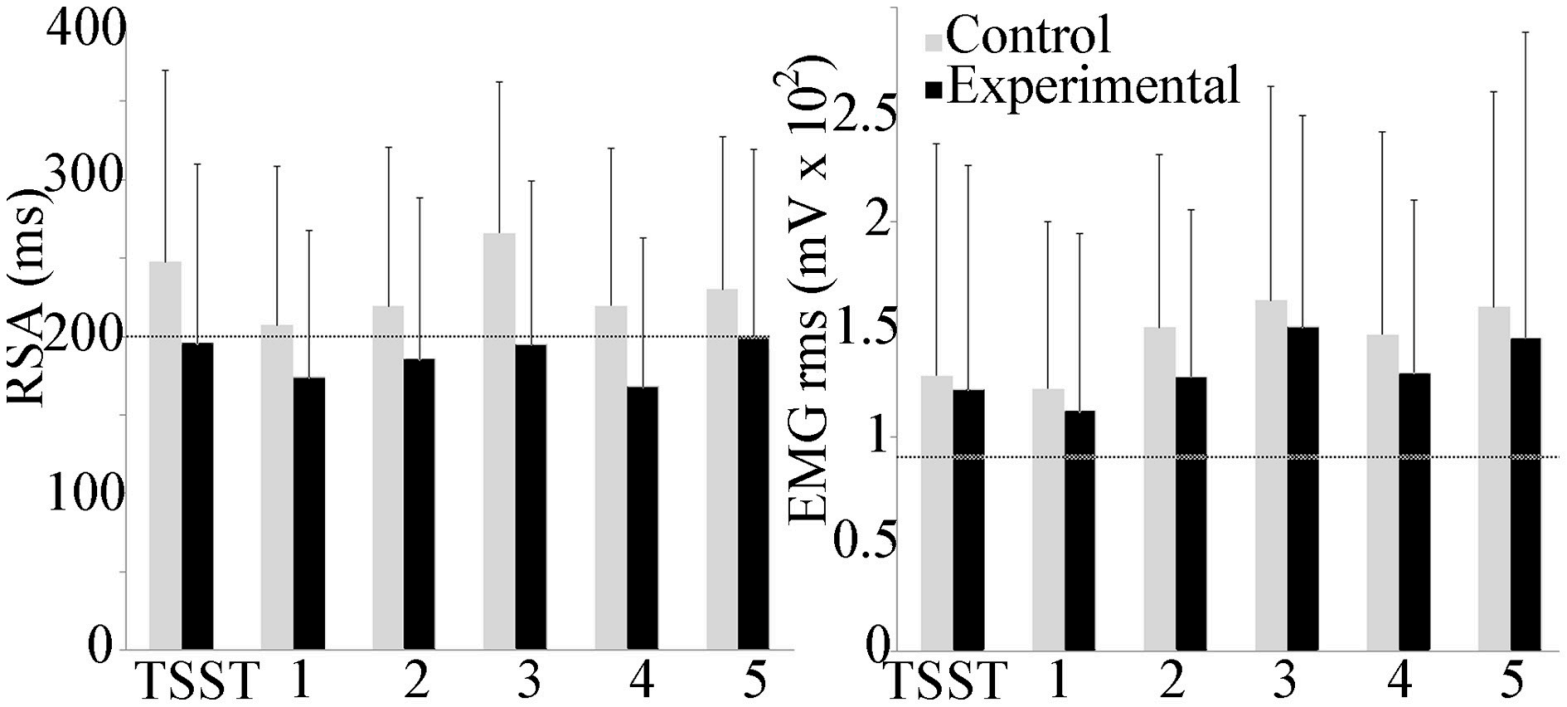
**Group differences in respiratory sinus arrhythmia (RSA) and trapezius muscle activity during the experiment**. Average baseline scores shown by dotted line. No group or scenario differences were observed.

Respiratory sinus arrhythmia (RSA) and mean trapezius EMG activity are shown in Figure 5. The experimental group trended lower than the control group throughout the experiment, but between group and scenario differences were not observed. Within groups, trapezius EMG activity trended higher than baseline activity throughout the study, with a significant increase in the control scenario 5 as compared to baseline (*p* ≤ 0.05). No group differences were observed with EMG activity.

### Resilience classification

Salivary cortisol was assessed immediately after the baseline and after the end of the experiment. At baseline, saliva cortisol did not differ between experimental and control groups, at a mean concentration of 0.38 (0.28) μg/dl. The final saliva cortisol sample, approximately 75 min following the end of the TSST, did not differ between experimental and control groups, with a mean concentration of 0.12 (0.08) μg/dl, which was significantly reduced as compared to initial concentration (*p* < 0.001).

**Figure 6:**
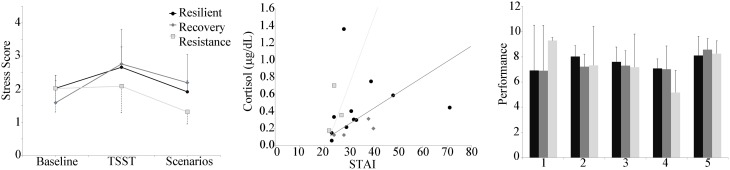
**Left panel:** Stress score by group plotted during the baseline, TSST, and throughout the 5 scenarios. **Center panel:** Decision boundaries of stochastic gradient descent linear classifier of resilience group using baseline cortisol and baseline STAI. **Right panel:** Performance by resilience group, showing a decrease in performance over the first 4 scenarios for the resistant group compared to much less variability in performance for the resilience and recovery groups.

Given that the above results indicated the scenarios did not provoke significant increases in physiological stress indices and served as more of a recovery phase than additional stressors, the pattern of these stress scores over the course of the experiment (baseline, socio evaluative stressor, VBS2 scenarios) was then analyzed to assess individuals' resilience to stress. Based on the responsivity of electrodermal and cardiovascular measures, a physiological stress score was defined for the experimental group only using a combination of heart rate and SCL, normalized by the mean control group values (Figure 6). As only the addition of the TSST in the experimental group provoked significant increases in physiological stress indices, only the experimental groups were used for the definition of resilience groups. Subjects were grouped by how their heart rate and skin conductance changed in response to the TSST and scenarios into the resistant, resilient, and recovery trends. (Norris et al., 2009) provided the basis for our definitions of the trends. One participant was removed due to loss of EDA. In the remaining participants, the majority of the individuals fell within the resilience phenotype (12/19), with 3 individuals in the resistant phentoype and 4 in the recovery.

Baseline cortisol was plotted against baseline STAI, with the participant data points marked according to the stress score trend type (Figure 6). At baseline, STAI reports for all participants was 30 ± 9. Two outliers (corresponding to resilient-trend subjects) were identified, one with a high cortisol level (1.36), and one with a high Baseline STAI (71). Leaving aside these two outliers, the distinct trend-groups clustered in a coherent way. The resistant-trend subjects tend toward having higher cortisol and lower STAI baseline values, the recovery-trend subjects tend toward having lower cortisol and higher STAI baseline values, and the resilience-trend subjects are scattered between the two groups. The classification algorithm whose decision bounds (Figure 6) rendered the best qualitative fit to this pattern was the stochastic gradient descent linear model method (using the default parameters, including an L2 penalty term weight with α = 0.0001, a hinge loss function, and the “optimal” learning rate setting). All of the 16 good non-outlier data points were used in the training due to the small sample size. The classification accuracy was 62.5%, with the recovery group having perfect classification, the resilient group having 4 points misclassified as Recovery and 1 misclassified as resistant, and the resistant group misclassifying 1 point as resilient.

In addition, the mean performance of the groups was assessed (Figure 6). Similar to the performance of the control group, the resistant group showed a decreased performance score over the first 4 scenarios. The resilient and recovery groups showed much less variability in performance score across the first 4 scenarios.

## Discussion

The ability to predict and measure an individual's resilience to stress will allow training practitioners to identify those in most need of additional preparation. This can aid in training to reduce physiological symptoms prior to task performance (Wood et al., 2007; Hourani et al., 2011) and can help in reduction of PTSD symptoms (Rothbaum et al., 2001). One of our goals in the experiment was to determine whether baseline measures taken prior to stressor delivery could be used to predict a subject's resilience to stressors, as measured by the trend of the subject's physiological reaction to the stressing conditions (in this case the socioevaluative stressor of the TSST). We grouped the subjects in the experimental condition into resistant-trend, resilient-trend, or recovery-trend. The resistant-trend subjects tend toward having higher cortisol and lower STAI baseline values, the recovery-trend subjects tend toward having lower cortisol and higher STAI baseline values, and the resilience-trend subjects are scattered between the two groups. This suggests a gradient of overall susceptibility (as measured by heart rate and skin conductance) to stressors that yields the smallest susceptibility (resistant-trend) when subjects perceive less stress than cortisol level indicates, and the greatest susceptibility (recovery trend) when perceived stress is greater than cortisol levels seem to indicate. Previous work has shown that age and aerobic fitness correlate strongly with hypothalamic-pituitary-adrenal axis (HPA) responses to stress (Traustadóttir et al., 2005; Rimmele et al., 2007; Webb et al., 2013), and the inclusion of a group with a greater variability in age and fitness scores would be expected to strengthen the diagnostic power of using baseline cortisol and self-reported stress to predict performance under stress.

The scatterplot and supervised learning-trained linear classifier analysis of baseline cortisol vs. perceived stress level (STAI) suggests that it may, in principle, be possible to classify stress response trends just using these measures, although a study is required with a larger sample size to determine if the observed pattern holds. The outliers observed may be due, respectively, to the subject just having woken up, since salivary cortisol levels tend to be highest within 20 min of waking (Tzortzi et al., 2009), and the subject recording a high baseline perceived stress rating due to a stressful event prior to the experiment. In the latter subject, heart rate and SCL data indicated a much stronger physiological stress response in the TSST vs. the baseline, calling into question the large baseline STAI value recorded. When outliers were ignored, the resistant-trend subjects tended to have higher cortisol and lower STAI, which means that their heart rate and skin conductance did not increase, despite sometimes high cortisol levels. A possible cause might be that these subjects have relatively high vagal tone, which has been associated with faster heart recovery from social stress (Souza et al., 2007). There is also evidence that cortisol secretion is higher in response to stress in subjects with high vagal tone (Smeets, 2011), which seems paradoxical, but may be a reflection of mechanisms designed to allow the sympathetic pathway to upregulate its effect in the face of consistently higher parasympathetic activity. Perhaps a combination of higher cortisol secretion and faster recovery could lead to baseline states of high cortisol and low perceived stress, such as we seem to find in the resistant-trend subjects.

The resilience- and recovery-trends were adjacent in the scatterplot and classifier feature space, with the least-recovery cases occurring when cortisol level was low and STAI high. One possibility may be that these subjects have low vagal tone, which has been associated with slower recovery of diastolic blood pressure after stress events (Weber et al., 2010). Less cortisol secretion may be required to trigger stress responses in these subjects, which could indicate how low cortisol/high perceived stress baselines might occur. In general, sympathetic nervous system activity leads to increased heart rate and sweating, whereas parasympathetic activity inhibits these (Everly and Lating, 2013). Therefore, subjects with higher baseline parasympathetic activity in general are likely to be more resilient to stress, and whereas lower baseline rates would render a subject more susceptible to sympathetic stress responses and slower to recover from the same.

The downward trend observed in the scenario performance of the resistance-trend group could indicate that these subjects were less engaged during the task and therefore both less concerned with poor performance (thus, lower stress during failure) and more inattentive to cues required to improve performance (thus, the failure to improve). Certain types of subjects, such persons diagnosed as having Antisocial Personality Disorder—manifest deficits in fear conditioning (Birbaumer et al., 2005) and a failure to learn from physical and social punishment cues (Schmauk, 1970). There may be some kind of “optimal middle” involving a subject's susceptibility to negative cues, where too little reaction leads to failure to improve performance and too much causes a level of distress that impairs cognitive flexibility and learning. There is evidence that heightened stress may improve memory consolidation, but impair memory retrieval (Smeets, 2011). Perhaps this could account for an inverse-U-shaped performance curve on some tasks requiring learning under stressful situations such as in a tactical situation.

One limitation of our trend analysis stems from a likely difficulty of distinguishing the recovery and resilience trends. Both of these show an increase, followed by a decrease; and it seems likely that the stress levels from the recovery trend would eventually reach the baseline stress levels after the experiment. Therefore, it may be that a better way to characterize these rise-and-fall trends would be to measure a stress-decay time constant, analogous to the time-constant on an exponential decay curve. Shorter time constants would indicate higher resilience and longer ones, less. Likewise, a stress rise time constant might measure the initial susceptibility if enough stress measures could be collected to discover where the stress is maximized.

Recent work has shown the utility of combining self-report and cortisol measurement in identifying depression risk (Owens et al., 2014). Using similar reasoning, here we show that cortisol assessment, combined with other physiological measures and self-reported stress can be useful in identifying individuals at risk for stress disorders. Whether such an approach has utility in identifying individuals at risk for mental illness is a subject of ongoing studies.

An additional goal of the experiment was to determine how virtual stress induction compares to and is influenced by the gold standard of socioevaluative stress. Here we show that VE stressors alone may not be sufficient to induce a significant stress response. However, results indicate the utility of adding stressors external to the virtual training environment to achieve a state of physiological stress, and that a relatively simple stress induction technique can have effects lasting many hours throughout a course of training. The use of the TSST significantly increased self-reported stress and electrodermal activity throughout training compared to control, while other physiological measures trended toward a difference without achieving statistical significance in this cohort. In a similar study using VBS2 scenarios with virtual stressors, participant stress increased both via self-report as well as physiological markers (Brouwer et al., 2011). The average STAI score from this group was 32, similar to what was observed in the control group of the current study. However, the experimental group that received the TSST had an average STAI score of 40 throughout the VBS2 scenarios, showing significantly more self-reported stress than studies that rely only on virtual stressors during training.

In contrast to the increased self-reported stress, electrodermal and cardiovascular reactivity in the experimental group, a number of measures did not significantly change with the addition of the socioevaluative stressor. Performance scores trended lower in each subsequent scenario as difficulty and stressors increased, with the exception of the final scenario. Several variables may have increased participant performance in the final scenario, including increased visual acuity and less complex simulation interaction (i.e., in the final scenario, the individual was merely trying to escape unharmed as opposed to laying explosives which required complex control interactions). Performance scores, which were calculated based upon success in meeting mission objectives, avoiding enemy fire and civilian casualties, as well as the scenario duration, did not differ between the experimental and control group, suggesting that the socioevaluative stressor was not sufficient to significantly impair performance in the experimental group.

Further, cortisol levels did not significantly increase after performance of the VBS2 scenarios. Due to the length of the study, the final cortisol assessment was approximately 75 min following the end of the socioevaluative stressor. The maximum cortisol response is expected to occur approximately 20–30 min following stress induction, with a linear decrease thereafter (Dickerson and Kemeny, 2004). Given that the VBS2 scenarios did not induce a significant physiological stress response as anticipated, the significant decrease in salivary cortisol observed across the course of the experiment is likely due to the natural decrease in cortisol secretion throughout the morning hours (Weibel et al., 1996).

In conclusion, this study explores stress induction, measurement, and the prediction of resilience to stress. The addition of a simple socio-evaluative stressor prior to simulation-based performance led to significant increases in physiological stress response and non-significant decreases in performance. Stress response was effectively captured via electrodermal and cardiovascular measures of heart rate and skin conductance level. Further, an algorithm which assesses changes in heart rate and SCL to quantify/qualify an individual's short term resilience to stress was developed and revealed the ratio of baseline perceived stress to baseline cortisol levels are potentially effective predictors of an individual's resilience to stress. Further research is needed to explore the effectiveness of such methods at predicting resilience. Continuing work is focused on adapting this training to military personnel, and assessing the utility of various coping and decision-making strategies on performance and physiological stress.

### Conflict of interest statement

The authors declare that the research was conducted in the absence of any commercial or financial relationships that could be construed as a potential conflict of interest.
